# The Determination of Cannabinoids in Urine Samples Using Microextraction by Packed Sorbent and Gas Chromatography-Mass Spectrometry

**DOI:** 10.3390/molecules27175503

**Published:** 2022-08-27

**Authors:** Luana M. Rosendo, Tiago Rosado, Patrik Oliveira, Ana Y. Simão, Cláudia Margalho, Suzel Costa, Luís A. Passarinha, Mário Barroso, Eugenia Gallardo

**Affiliations:** 1 Centro de Investigação em Ciências da Saúde, Universidade da Beira Interior (CICS-UBI), Av. Infante D. Henrique, 6201-506 Covilhã, Portugal; 2 Laboratório de Fármaco-Toxicologia, Ubimedical, Universidade da Beira Interior, Estrada Municipal 506, 6200-284 Covilhã, Portugal; 3 Serviço de Química e Toxicologia Forenses, Instituto de Medicina Legal e Ciências Forenses-Delegação do Centro, 3000-213 Coimbra, Portugal; 4 Serviço de Química e Toxicologia Forenses, Instituto de Medicina Legal e Ciências Forenses-Delegação do Sul, 1169-201 Lisboa, Portugal; 5 UCIBIO-Apllied Molecular Bioesciences Unit, Departamento de Química, Faculdade de Ciências e Tecnologia, Universidade NOVA de Lisboa, 1099-085 Caparica, Portugal; 6 Associate Laboratory i4HB-Institute for Health and Bioeconomy, NOVA School of Science and Technology, Universidade NOVA, 2819-516 Caparica, Portugal

**Keywords:** cannabinoids, Δ^9^-tetrahydrocannabinol, urine, microextraction by packed sorbent, gas chromatography-mass spectrometry

## Abstract

Cannabis is the most consumed illicit drug worldwide, and its legal status is a source of concern. This study proposes a rapid procedure for the simultaneous quantification of Δ^9^-tetrahydrocannabinol (THC), 11-hydroxy-Δ^9^-tetrahydrocannabinol (11-OH-THC), 11-nor-9-carboxy-Δ^9^-tetrahydrocannabinol (THC-COOH), cannabidiol (CBD), and cannabinol (CBN) in urine samples. Microextraction by packed sorbent (MEPS) was used to pre-concentrate the analytes, which were detected by gas chromatography–mass spectrometry. The procedure was previously optimized, and the final conditions were: conditioning with 50 µL methanol and 50 µL of water, sample load with two draw–eject cycles, and washing with 310 µL of 0.1% formic acid in water with 5% isopropanol; the elution was made with 35 µL of 0.1% ammonium hydroxide in methanol. This fast extraction procedure allowed quantification in the ranges of 1–400 ng/mL for THC and CBD, 5–400 ng/mL for CBN and 11-OH-THC, and 10–400 ng/mL for THC-COOH with coefficients of determination higher than 0.99. The limits of quantification and detection were between 1 and 10 ng/mL using 0.25 mL of sample. The extraction efficiencies varied between 26 and 85%. This analytical method is the first allowing the for determination of cannabinoids in urine samples using MEPS, a fast, simple, and low-cost alternative to conventional techniques.

## 1. Introduction

Cannabis is one of the oldest and most commonly abused narcotics, and its legal status is currently a source of global concern [1]. Several preparations of *Cannabis sativa*, including marijuana, are consumed by 200–300 million people worldwide, according to the United Nations Office on Drugs and Crime (UNODC), making them the most popular illicit substances of the twenty-first century [2,3].

Changes in laws such as legalization or decriminalization in nations such as the United States, Canada, and Portugal contribute to its problematic use [4]. Legalized cannabis is still causing concern for several reasons: There is evidence that cannabis has a detrimental effect on the neuronal connections of the developing brain, requests for dependence treatment are increasing worldwide, and cannabis intoxication poses several dangers, especially when driving [2,5]. According to the most recent European School Survey Project on Alcohol and Other Drugs (ESPAD) report from 2019, around 4% of students in the whole ESPAD population are at risk of developing cannabis-related problems [6]. Since the prevalence of cannabis has increased year after year, it is expected that a greater number of people will need therapy. In Europe, over 111 000 people sought specialized drug treatment for problems related to cannabis use in 2019 (35% of all treatment requests); of these, approximately 62 000 sought therapy for the first time [7].

The recreational use of marijuana impairs cognitive and psychomotor performance and can also alter judgment and cause paranoia or psychosis at high doses [8,9,10]. Therefore, health authorities should be particularly aware of these effects, especially now that decriminalization and/or legalization of use is being adopted in multiple countries. Nonetheless, cannabis may be also used for medicinal purposes, normally for pain control and in neurodegenerative disorders (e.g., Parkinson’s, Alzheimer’s, and multiple sclerosis), oncology patients to minimize symptoms such as nausea and vomiting, and other disorders as well [2,11,12].

The most analyzed cannabinoids in clinical toxicology are Δ^9^-tetrahydrocannabinol (THC), which is the most psychoactive of the cannabinoids listed; 11-hydroxy-Δ^9^-tetrahydrocannabinol (11-OH-THC), a hallucinogenic metabolite obtained by microsomal hydroxylation of THC; the inactive substance 11-nor-9-carboxy-Δ^9^-tetrahydrocannabinol (THC-COOH), a product of the oxidation of 11-OH-THC with great interest for diagnostic purposes; cannabidiol (CBD), also a prevalent cannabinoid in marijuana plants; and cannabinol (CBN). These last two cannabinoids have no known psychoactive properties [2,13,14,15].

Proper sample preparation is necessary for gas chromatography–mass spectrometry (GC–MS) analysis, as it has a significant impact on analyte isolation from complex matrices, including urine. Urine has similar distribution and higher detection windows in comparison with blood and oral fluid but requires a simpler pre-treatment [16] This sample is widely used in testing for driving under the influence of drugs (DUID) and workplace drug testing [16,17].

Cannabinoids may be identified in the urine for days or weeks after usage due to their high tissue retention depending on the frequency and length of exposure [2,15,16].

Microextraction by packed sorbent (MEPS) is a miniaturization of solid-phase extraction (SPE) that is mostly applied for drug measurements [18,19,20,21]. The main distinction between the two techniques is that in MEPS, the sorbent material is inserted directly into a syringe rather than being placed in a separate column as occurs in SPE. Additionally, the quantities of solvents and samples are significantly reduced in MEPS [18,19,20,21]. This approach, in combination with techniques like GC–MS, is a powerful tool for screening and for the determination of several compounds in biological samples [19,20,22,23,24,25,26,27,28]. Additionally, MEPS can be fully automated when coupled online, without any adjustments [20,29,30].

This extraction technique has been used to identify and quantify THC and metabolites in oral fluid samples using liquid chromatography–tandem mass spectrometry (LC-MS/MS) [31]. When 50 mM of ammonium hydroxide (NH_4_OH) in methanol was used as the elution solvent, the authors provided a straightforward extraction process that involved 5 sample strokes and yielded recoveries from 50 to 100% [31]. Rosado et al. [32] applied MEPS to plasma samples, the most commonly used sample for drug monitoring, also with the goal of extracting cannabinoids. The chosen elution solvent was 10% NH_4_OH in methanol, and the authors were able to achieve recoveries from 50 to 70% [32]. Furthermore, Sartore et al. [33] quantified THC and metabolites in urine samples by combining LC-MS/MS with MEPS using molecularly imprinted polymers (MIPs) as sorbent [33]. The procedure was improved by using 10 sampling strokes and 4 cycles of elution with 90% acetonitrile solvent [33]. However, to the best of our knowledge, CBD, CBN, THC, and metabolites have not yet been quantified in urine samples using MEPS. Taking into account this situation, and the known advantages of MEPS (lower solvents and sample volumes, possibility of reusing the same cartridge several times), the goal of this work was to develop and optimize the first application of MEPS in combination with GC–MS for the identification and quantification of cannabinoids in urine samples. Its application will result in a simple, rapid, sensitive, and less expensive method of determining the target analytes.

## 2. Results and Discussion

### 2.1. Optimization of the Extraction Procedure

#### 2.1.1. Extraction Procedure Selection

The initial stage of this experiment was to choose the best extraction procedure. According to their unique features and available literature [18,19], six different extractions were tested.

The first approach was based on that by Rosado et al. [32], a procedure used to extract THC and main metabolites from plasma samples. A number of modifications were made to the elution step, which employed 6 cycles of 100 µL of 2% NH_4_OH in methanol. The reconstitution of the sorbent was modified as well, and the following conditions were used: 2 cycles of 250 µL of 1% NH_4_OH in methanol: acetonitrile (1:1), then 2 cycles of 250 µL of 1% formic acid in isopropanol: water (10:90). Following unsuccessful experiments, it was decided to try another method based on that by Simão et al. [26] that used the same biological specimen, again with unsatisfactory outcomes.

The tutorial published by Abdel-Rehim [18] served as a guide for the final methodology. This protocol was amended twice before choosing the final solutions, and all suggested steps were maintained except those that follow. (1) washing step: 1 cycle of 100 µL of 0.1% of formic acid in water and (2) elution step: 100 µL 0.1% of NH_4_OH in methanol. However, the results were unsatisfactory when compared with the original approach by Abdel-Rehim, from which higher analyte recoveries were obtained. As a result, the method that was chosen for additional optimization included the steps that follow: conditioning with 50 µL of methanol and 50 µL of water; sample load with five draw–eject cycles of 150 µL; sorbent wash using 100 µL of 0.1% formic acid in water with 5% isopropanol, followed by 2 air strokes (150 µL); elution with 50 µL of 0.1% of ammonium hydroxide in methanol.

#### 2.1.2. Optimization of the Experimental Design

The design of experiments (DOE) statistical tool allows for assessing in a multivariate fashion the critical factors that have a significant impact on the extraction procedure. Consequently, it leads to higher target analyte recoveries, and it is the ideal tool when optimization is required. The studied factors for this method were: number of sample draw–eject cycles (strokes); number of washes; and number of elutions.

**Figure 1:**
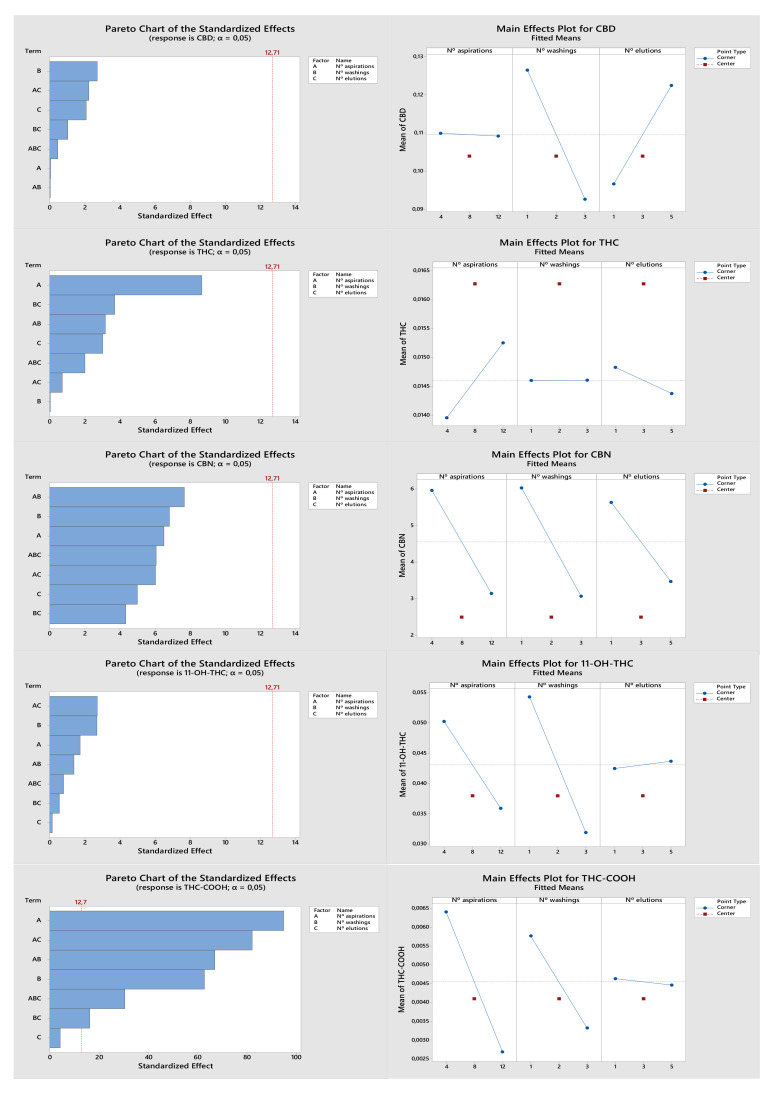
Graphical representation of DOE optimization for CBD, THC, CBN, 11-OH-THC, and THC-COOH.

Figure 1 shows the pareto and main effects charts obtained with the DOE analysis for each target analyte. All analytes have different pareto charts, but none of the evaluated parameters (factors with influence on analyte recovery) had a significant influence on the response, except for THC-COOH, for which all factors showed a significant influence. In the main effects plots, we observe the individual effect of each factor under study. A lower number of sample draw–eject cycles (4 × 150 µL) results in greater recoveries for most of the target analytes except THC. Regarding the number of sorbent washes, a single wash (1 × 50 µL) also gave a better response for all analytes except CBN. Finally, the number of elutions varied for each analyte. While better recoveries were obtained with a lower number of elutions (1 × 100 µL) for THC, CBN and THC-COOH, a greater number of elutions (5 × 100 µL) appeared as the most suitable for CBD and 11-OH-THC. Overall, one should consider that all these factors had no significant influence on the response for most cannabinoids, and only THC-COOH was markedly influenced. In addition, THC-COOH is the main metabolite in urine samples and the most important for cannabis consumption confirmation. Since all factors revealed a significant influence on THC-COOH and interactions between factors also appear to play a significant role on its recovery, it was important to optimize the method further.

**Figure 2:**
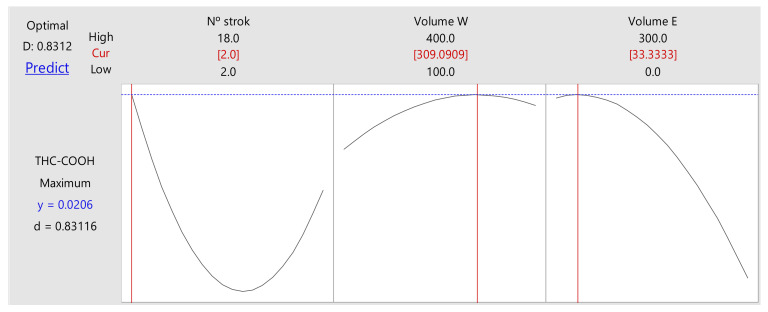
Results from RSM response optimizer for THC-COOH. N^0^ strok: number of strokes; Volume W: washing volume; Volume E: elution volume.

The experimental response surface methodology (RSM) was applied for this compound. A new matrix containing the same variables was constructed (number of strokes, washing volume, and elution volume). The optimal response (Figure 2) resulted in 2 cycles for sampling, 309 µL of washing solution and 33 µL of elution solution; the values were rounded to 310 µL and 35 µL, respectively.

With this last optimization step, the final MEPS procedure was as follows: sorbent conditioning with 50 µL of methanol and 50 µL of water, sample load with two draw–eject cycles, washing with 310 µL of 0.1% formic acid in water with 5% isopropanol followed by 2 air strokes (150 µL), and finally, the elution with 35 µL of 0.1% ammonium hydroxide in methanol.

### 2.2. Method Validation Parameters

#### 2.2.1. Selectivity

**Figure 3:**
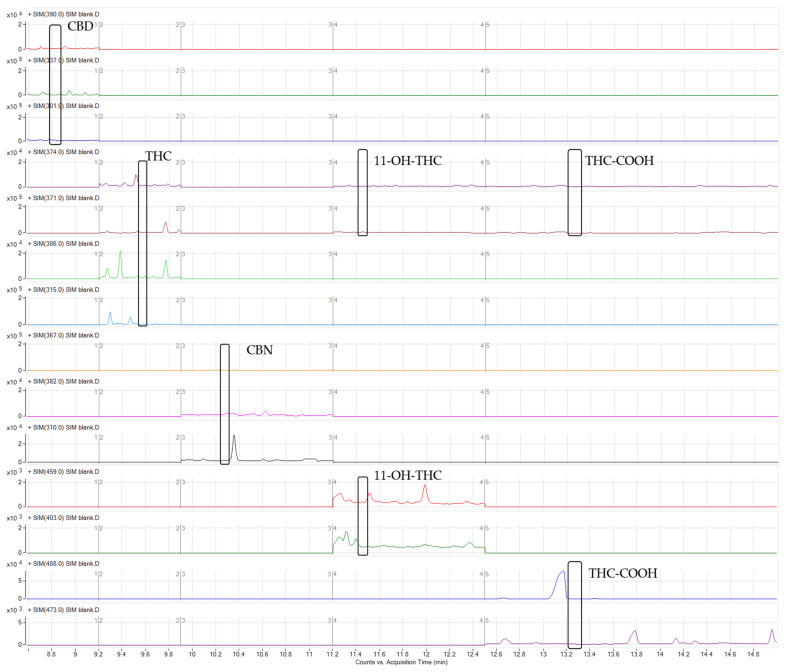
Chromatogram of a blank sample.

The selectivity of the method demonstrates its capacity to detect the compounds under study in the presence of additional interference from the biological samples analyzed [34,35]. Drugs and endogenous components that produce similar high molecular weight ions may potentially interfere if their retention times are similar and unsuitable ions are chosen for monitoring. For this reason, the selectivity of the method was assessed by analyzing ten distinct blank urine samples collected from laboratory staff members who did not consume cannabis. Additionally, three typical molecular weight ions for each analyte were chosen carefully, where no matrix or endogenous interferences were observed, as can be observed in a blank sample (Figure 3).

#### 2.2.2. Linearity and Calibration Model

The procedure was linear for THC and CBD from 1 to 400 ng/mL, for CBN and 11-OH-THC from 5 to 400 ng/mL, and for THC-COOH from 10 to 400 ng/mL. To account for heteroscedasticity, weighted least squares regressions were used. Each compound underwent evaluation using six weighting factors (1/√x, 1/x, 1/x^2^, 1/√y, 1/y, 1/y^2^), and the weighting factor that produced the best results was selected [35]. The factor with the lowest sum of errors and a mean R^2^ of at least 0.99 was selected (Table 1). Using these weighted least squares regressions, the current analytical approach was linear with a calibrator’s accuracy (mean relative error (bias) between the measured and spiked values) within a ±15% range for all concentrations. Coefficients of variation (CVs) typically lower than 15% were obtained for precision. Table 1 displays calibration data.

#### 2.2.3. Limits of Detection and Quantification

**Figure 4:**
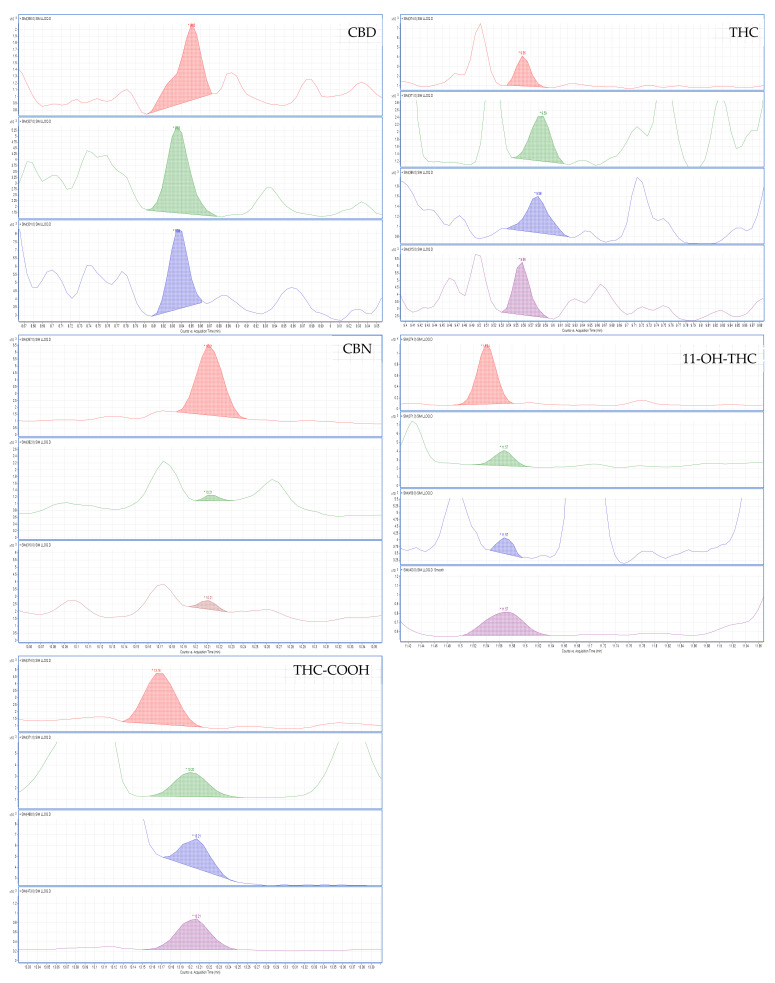
Ion chromatogram of a spiked sample at the LLOQ (1 ng/mL for CBD and THC, 5 ng/mL for CBN and 11-OH-THC, and 10 ng/mL for THC-COOH).

According to the guidelines of the Substance Abuse and Mental Health Services Administration (SAMHSA) [36] and European guidelines for workplace drug testing in urine (EWDTS) [37], the cut-off for cannabis metabolites for laboratory screen tests is 50 ng/mL. The confirmation test cut-off concentration is recommended to be 15 ng/mL for THC-COOH. In this sense, our method can be considered suitable for cannabinoid determination in urine samples since it achieves lower limits of quantification (LLOQ) from 1 to 10 ng/mL. The LLOQ obtained for CBD and THC was 1 ng/mL; for CBN and 11-OH-THC, 5 ng/mL; and for THC-COOH, 10 ng/mL. The detection limits (LOD), on the other hand, were 1 ng/mL for CBD, THC, and 11-OH-THC and 5 ng/mL for CBN and THC-COOH (Table 1). As a result, the LLOQs are quite satisfactory (Figure 4), especially when compared with other published analytical techniques for the same analytes.

The current method achieves lower or similar LODs than the literature for: CBD [38,39,40,41,42]; THC [33,38,39,40,41,42,43,44,45]; CBN [41]; 11-OH-THC [33,38,39,40,41,42,43,44]; and THC-COOH [38,46,47]. The same LLOQs are reported for: CBD [39,40,41,42,48,49]; THC [33,39,40,41,42,43,44,45,48,49]; CBN [39,40,41,48,49]; 11-OH-THC [33,39,40,41,48,49]; and THC-COOH [41,46,47,48,49]. When using a GC–MS system [41,43,44], the authors report greater LODs and LLOQs than those obtained with the herein proposed technique. The same happens when using systems with greater sensitivity, such as LC–MS/MS [38,39,40,42,45,46,47,48,49]. Nonetheless, some authors report lower LLOQs than those obtained with our MEPS–GC–MS, mostly using techniques with greater sensitivity, including GC–MS/MS [50,51,52,53] and LC–MS/MS [38,39,42,46,54,55,56,57,58,59]; several of these approaches also used larger sample volumes. Table 2 summarizes the information.

The advantages of this novel technique can be deduced by comparing the obtained limits as well as the reduced preparation times, reduced solvent volumes (microlitres), and reduced sample volume requirements. The current approach is the first to rapidly detect and quantify cannabinoids in urine samples using MEPS and GC–MS, requiring only 250 µL of sample and reaching LLOQs that may be deemed appropriate according to the literature, which can be considered quite attractive for implementation in routine forensic toxicology analysis.

**Figure 5:**
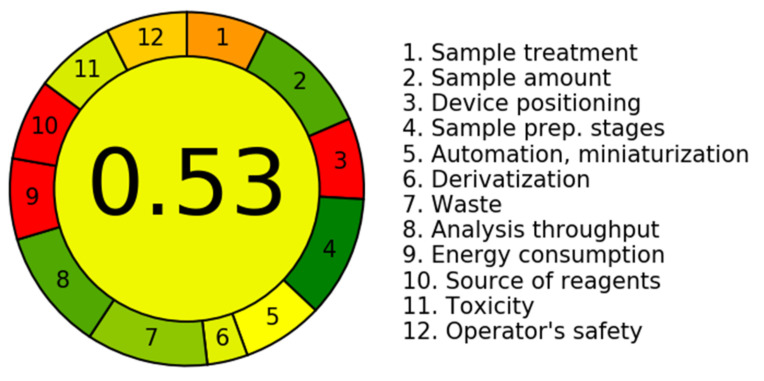
Method evaluation according to the AGREE-Analytical GREEnness Metric Approach.

This method was classified according to the according to the AGREE-Analytical GREEnness Metric Approach, in which all its steps are individually evaluated concerning their greenness [61,62]. Considering this classification, the main limitations of this method are the need for sample pretreatment and the fact that it involves manual operation (Figure 5).

#### 2.2.4. Intra-Day, Inter-Day, and Intermediate Precision and Accuracy

Intra-day precision and accuracy were evaluated by analyzing five different concentration levels within the linearity range (n = 5). The obtained CVs were lower than 15% at all studied concentrations, with a mean relative error within ±14% (Table 2).

The evaluation of inter-day precision and accuracy was made within a 5-day period for all calibrators. The obtained CVs were lower than 15% for all analytes at all tested concentrations, with an inaccuracy within ±15% (Table 3).

To assess intermediate precision and accuracy, 3 quality control samples (QCs) with concentrations of 15, 240, and 360 ng/mL were evaluated during a period of 5 days (n = 3). The obtained CVs were typically lower than 13% with an inaccuracy within ±10% (Table 3).

#### 2.2.5. Extraction Efficiency

To assess extraction efficiency, 2 sets of samples (n = 3) were prepared by spiking blank urine with the target analytes at 3 different concentrations: 50, 100, and 400 ng/mL. The first set represented pre-extraction spiked samples, while the second set represented post-extraction spiked ones (corresponding to 100% recovery). To calculate extraction efficiency, a ratio was calculated between the relative peak areas of sample set 1 with those of sample set 2. Table 4 displays the target analytes’ extraction efficiencies obtained with the optimized MEPS procedure.

The herein described method resulted in greater extraction efficiencies when compared with the only known publication using MEPS in urine for screening THC and metabolites [11]. Using MIP sorbent, the authors determined THC and the major metabolites in urine samples and obtained recoveries between 3 and 18% [11].

Micro-solid-phase extraction (µ-SPE) [39], molecularly imprinted solid-phase extraction (MISPE) [43,45], disposable pipette extraction (DPX) [42,55,63], dispersive liquid–liquid microextraction (DLLME) [53,60], packed in-tube solid-phase microextraction (IT-SPME) [48], and hollow fiber membrane solvent microextraction (HFMSME) [64] are other miniaturized sample preparation techniques that have been applied to urine samples to extract cannabinoids. However, extraction efficiencies were not reported for some of the latter, namely DPX [63], SMPE [48], and DLLME [53].

The authors who used µ-SPE [39] obtained recoveries ranging from 65 to 85% for all analytes, the lower being CBD (65–69%) and the highest for CBN (80–85%). Montesano et al. [39] obtained greater recoveries when compared with those here described, although the values obtained for THC-OH and THC-COOH were similar [39]. Nestić and colleagues [43] compared two extraction techniques, non-imprinted polymers (NIP) and MIP, both for SPE. The results for NIP were less favorable than those for MIP [43]. In comparison with the results obtained in the present work, NIP resulted in lower recoveries for the main THC metabolites [43]. However, MIP extraction efficiency was similar to ours [43]. THC extraction efficiencies in both procedures were greater than those obtained with our MEPS procedure [43]. For instance, Lendoiro et al. [45] report similar recoveries to ours for THC (26.6–34.5%) but lower for THC-COOH (15.9–32.4%) when MISPE was used to extract THC and THC-COOH from urine samples. Andersson et al. [55] opted for DPX using WAX-S tips. The extraction efficiencies for the compounds varied: CBD (68.7–73.6%); CBN (54.7–52.3%); THC (55.7–58.4%); 11-OH-THC (71.1–73.6%); and THC-COOH (69.4–73.9%) [55]. The recoveries in the latter were comparable with those published by Sempio et al. [42], who used the same tips. Greater recoveries were obtained for THC-COOH (85.6–88.5%) and smaller ones for THC (44.3–46.9%) [42].Using surfactant-assisted dispersive liquid–liquid microextraction (SA–DDLME), the values obtained for CBD, CBN, and THC ranged between 47.5 and 73.0% [60]. Finally, for HFMSME, the recoveries of THC-COOH were in a 2.6–4.5% range [64].

Even though our MEPS procedure may present lower recoveries than some of the techniques studied by other authors, it is possible to affirm that the MEPS-optimized procedure is quite efficient for this purpose since LLOQs below the recommended cut-offs were obtained with low sample volumes.

#### 2.2.6. Stability

The stability of the target analytes was studied under specific conditions and time intervals to mimic those usually used for collection and storage of biological samples. In this study, short-term stability, stability following freeze/thaw cycles, and stability in processed samples were evaluated to assess the behavior of the analytes.

The evaluation of the stability in processed samples was conducted at the same concentrations as the QC samples (n = 3), in which previously analyzed samples were reanalyzed after kept in the autosampler for 24 h. The analyte concentrations were determined based on the original calibration curve, obtaining CVs between 0.12 and 14.20% and a mean RE within a ±13.40% interval (Table 5).

Short-term stability was also assessed in triplicate for blank urine samples spiked at the QC concentration levels. For this evaluation, urine samples were spiked and maintained at room temperature for 24 h. These samples were then extracted with our MEPS procedure and compared with freshly prepared samples. The CVs obtained were lower than 10.00% for all the target analytes, while the mean RE was within ±15.00%.

The freeze/thaw stability was investigated at the same three concentration levels (n = 3). The spiked samples were frozen at −20 °C for 24 h, and then they were allowed to thaw unassisted at room temperature. The samples were then re-frozen for 24 h, completing one cycle. Three freeze/thaw cycles were performed in total, after which the samples were extracted, analyzed, and compared with samples prepared and tested the same day. All compounds were considered stable in urine after the three cycles of freeze/thaw, considering that the CVs achieved were lower than 10.00% and the mean RE was within a ±14.00% interval.

In all evaluation stability tests, all analytes were deemed stable in urine (Table 5).

#### 2.2.7. Carryover

Carryover was evaluated by injecting two different solvents used for sorbent reconstitution: (1) 400 µL of 0.1% NH_4_OH in methanol: acetonitrile (1:1) and (2) 250 µL of methanol. Before the washing for the re-use of the sorbent, a sample spiked at the upper limit of quantification (ULOQ) was extracted. The solvents were submitted to the same process of evaporation and derivatization. Since there were no signals in the retention time or selection ions for the target analytes, we can conclude that no carryover effect exists between extractions with the same MEPS sorbent.

#### 2.2.8. Method Applicability

The present method was successfully applied to seven authentic urine samples obtained from cannabis consumers who voluntarily accepted to participate in the study (Table 6).

## 3. Materials and Methods

### 3.1. Reagents and Standards

The analytical standards of THC, 11-OH-THC, THC-COOH, CBD, and CBN and the internal standards (ISs): Δ^9^-tetrahydrocannabinol-d_3_ (THC-d_3_); 11-hydroxy Δ^9^-tetrahydrocannabinol-d_3_ (11-OH-THC-d_3_); and 11-nor-9-carboxy-Δ^9^-tetrahydrocannabinol-d_3_ (THC-COOH-d_3_) were purchased from Sigma Aldrich (Sintra, Portugal). Methanol, isopropanol, ethyl acetate, acetonitrile, and glacial acetic acid (99%) were obtained from Fisher Scientific (Loughborough, UK) and were all of HPLC grade except the latter (analytical reagent grade). Formic acid (99–100%) was obtained from Chem-Lab (Zedelgem, Belgium), sodium hydroxide from LabChem (Santo Antão do Tojal, Portugal), and ammonium hydroxide from Enzymatic (Santo Antão do Tojal, Portugal). N-Methyl-N-(trimethylsilyl) trifluoroacetamide (MSTFA) and trimethylchlorosilane (TMCS) were provided by Macherey-Nagel (Düren, Germany). Deionized water (resistivity 18.2 MΩ·cm at 25 °C and total organic carbon ≤5 ppb) was obtained from a Milli-Q System (Millipore, Billerica, MA, USA). The MEPS syringe (250 µL) and M1 cartridges (4 mg; 80% C_8_ and 20% SCX) were purchased from SGE Analytical Science (Victoria, Australia).

Stock solutions of each analyte were prepared at 100 µg/mL by proper dilution with methanol. Working solutions for THC, 11-OH-THC, THC-COOH, CBD, and CBN were prepared by diluting stock solutions with methanol to a final concentration of 10 µg/mL, and a working solution of IS at 100 ng/mL was also prepared in methanol. All solutions were kept at 4 °C in the absence of light.

### 3.2. Biological Specimens/Urine Samples

The drug-free urine samples used in all experiments were provided by laboratory staff. These samples were stored at −20 °C.

Authentic urine samples were kindly provided by students of Universidade da Beira Interior (UBI) after reading and accepting an informed consent (Ethical Committee project: CE-UBI-Pj-2022-035-ID1349) and were stored at −20 °C until analysis. The criteria for inclusion were based on recent reports. The age range was between 15 and 34 years, which includes university students, and this population has the largest percentage of cannabis users according to the EMCDDA [65].

### 3.3. Gas Chromatographic and Mass Spectrometric Conditions

For the chromatographic analysis, an HP 7890B gas chromatographic system was used together with an Agilent Technologies 5977A mass spectrometer and an Agilent 7693 autosampler. For the separation of the analytes, a capillary column (30 m 0.25-mm I.D., 0.25 µm film thickness) with 5% phenylmethylsiloxane (HP-5MS) provided by J & W Scientific (Folsom, CA, USA) was used.

The oven temperature started at 150 °C, holding for 2 min, followed by an increase of 20 °C/min during 5 min until 270 °C, after which a second temperature ramp was performed with increases of 15 °C/min until a temperature of 300 °C was reached. In splitless mode, 3 µL of the derivatized extract was injected, with inlet, transfer line, and detector temperatures set at 220 °C, 230 °C, and 280 °C, respectively. The carrier gas (helium) flow rate was kept constant at 1 mL/min, and data were acquired in the selected ion monitoring (SIM) mode. A filament current of 35 A and an electron energy of 70 eV were adopted.

The quantifier and qualifier ions used to monitor each analyte, as well as their respective retention times, are presented in Table 7.

In order to evaluate the retention times of the compounds, a mixture of the compounds and IS was injected in each run before each batch of analysis; in addition, relative retention times were evaluated and used for compound identification. Linear hydrocarbons were not used in this study to evaluate linear retention indices.

### 3.4. Sample Preparation

Frozen urine samples were thawed at room temperature and centrifuged for 15 min at 4500 rpm before analysis. Authentic urine samples were hydrolyzed before extraction according to the literature [31,51,55,56,57,58]. Hydrolysis was performed by mixing urine (250 µL) with 10 µL of 10 M NaOH and incubating for 15 min at 60 °C. After this cooled to room temperature, 100 µL glacial acetic acid was added and then vortex mixed. Lastly, 20 µL of the IS working solution at 100 ng/mL was added.

The MEPS procedure was fully optimized (Section 3.5), and the final conditions were as follows. (*i*) M1 sorbent condition with 50 µL of methanol and 50 µL of water; (*ii*) sample load with two draw–eject cycles of 150 µL; (*iii*) sorbent wash using 310 µL of 0.1% formic acid in water with 5% isopropanol, followed by two air strokes (150 µL) to dry the sorbent; (*iv*); and finally the retained analytes were eluted with 35 µL of 0.1% ammonium hydroxide in methanol. The obtained extract was subsequently evaporated to dryness under a gentle stream of nitrogen. The dried extracts were derivatized in a microwave oven (800 W) for 2 min with 40 µL of MSTFA containing 5% TMCS, and a 3 µL aliquot of the resulting solution was injected into the GC–MS equipment.

A last step was added after each extraction in order to reconstitute the sorbent for the next extraction. The sorbent reconstitution was made with two solutions: 0.1% of ammonium hydroxide in methanol: acetonitrile (1:1) and 0.1% formic acid in isopropanol: water (9:1) (four cycles of 100 μL, each) [18].

### 3.5. MEPS Procedure Optimization

It is of utmost importance to optimize the extraction procedure to eliminate interference and enhance extraction efficiency. A total of six different approaches were considered to evaluate washing and elution solvents, selected based on the available literature [18,19] and the physical-chemical properties of the target analytes.

The Design of Experiments (DOE) approach was used for the further optimization of the MEPS procedure. A two-level, three-component factorial design (2^3^) was used to screen the factors (independent variables), with considerable relevance of the recovery of the target analytes as well as their major effects. These factors were determined to be the number of sample draw–eject cycles (strokes), the number of washing cycles or the total volume, and the volume or the number of elution cycles [18,19,20,30]. A total of eleven runs (treatment combinations) were required to cover all potential combinations of factor values. As follows, the independent variables were investigated at 2 levels (low and high): number of strokes (4 and 12); number of washings (1 and 3); and number of elutions (1 and 5). To reduce the influence of noise factors and minimize systematic errors, these experiments were conducted in a random order with a center point (n = 3).

Further optimization was applied using response surface methodology (RSM), since the above experimental design did not allow for drawing a proper conclusion due to interactions between components, namely for THC-COOH, for which interactions had a significant influence. A new matrix containing the same variables (number of strokes, amount of washing, and number of elutions) was constructed. The number of strokes ranged from 1 to 15, the volume of washing solvent ranged from 150 µL to 450 µL, and the volume of elution solvent ranged from 50 µL to 250 µL.

### 3.6. Validation Procedure

The described method was fully validated according to the guiding principles of the Scientific Working Group for Forensic Toxicology (SWGTOX) [34] and the Food and Drug Administration (FDA) [35]. The studied parameters included selectivity; linearity and limits; intraday, interday, and intermediate precision and accuracy; extraction efficiency; stability; and carryover.

Spiked samples were prepared and analyzed using the MEPS extraction approach. The linearity of the method was determined on these spiked samples (n = 5) in the range of 1–400 ng/mL.

Calibration curves were obtained by plotting the peak area ratio between each analyte and the IS against analyte concentration. Acceptance criteria were: a determination coefficient (R^2^) of at least 0.99; accuracy within ±15% (excluding the LLOQ); and CVs equal or lower than 15% (excluding the LLOQ).

The LLOQ was established as the minimum concentration that could be measured with acceptable accuracy and precision, i.e., with a relative error (RE) of less than ± 20% of the nominal concentration and a coefficient of variation (CV, percent) lower than 20%. The limits of detection (LOD) were obtained by examining five repetitions of spiked samples and were defined as the lowest concentrations that produced a distinct peak that was clearly discernible from the blank and had a signal-to-noise ratio of at least 3 [34,35].

Five replicates of blank urine samples spiked with the target analytes at a minimum of four different concentration levels were examined on the same day in order to assess intraday precision and accuracy. Within a five-day span, interday precision and accuracy were assessed at a minimum of six concentrations. Intermediate precision and accuracy were evaluated with three QC samples (n = 3) at the concentrations of 15, 240, and 360 ng/mL along the 5-day protocol [34,35].

For the analysis of extraction efficiency, 2 sets of samples (n = 3) were prepared at 3 concentration levels (50, 100, and 400 ng/mL). Set one represented pre-extraction spikes, while set 2 consisted of post-extraction spikes (representing 100% efficiency). The IS was added to both sets of samples after extraction. The efficiency results were obtained by the ratio between the relative peak areas of sample set 1 with those of sample set 2.

A total of 3 concentration levels corresponding to the QCs concentrations (15, 240, and 360 ng/mL) (n = 3) were evaluated to study the target analytes stability under different conditions (processed samples, short-term stability, and freeze/thaw stability). To evaluate the processed samples’ stability, the previously analyzed extracts were re-analyzed after being stored at room temperature in the autosampler for 24 h, and their concentrations were measured using the original calibration curve. In order to test for short-term stability, blank samples were spiked and kept at room temperature for 24 h. These samples were then extracted and compared with freshly prepared samples. In order to test for freeze-thaw stability, urine samples were spiked and kept at −20 °C for 24 h. Following this time, the frozen samples were thawed unassisted at room temperature before being refrozen for another 24 h under the same conditions. Following the third cycle of this freeze/thaw process, the samples were extracted, analyzed, and compared with samples prepared and tested the same day [34,35]. The analyte was deemed stable for each stability study if the CV between the two sets of samples was under 15%.

The carryover was analyzed after extracting the sample spiked at the ULOQ. The solvents used to evaluate the carryover effect were the same ones use for the reconstitution of the MEPS sorbent. A carryover effect is observed when signals appear at the selected ions and retention time of the target analytes [34,35].

## 4. Conclusions

A novel method was developed and optimized for the determination of selected cannabinoids in urine samples using MEPS and GC-MS. In order to maximize the analyte recovery and thus obtain lower detection and quantification limits, the method was carefully optimized using the experimental design approach. The optimized MEPS procedure proved to be simple, quick, sensitive, and accurate. The proposed analytical method was successfully validated and applied to authentic samples.

The current analytical method requires only 250 µL of urine and has been demonstrated to be linear within the adopted concentration ranges for each analyte and allowed to reach LLOQs between 1 and 10 ng/mL. The proposed method can be viewed as extremely advantageous, user-friendly, and economically appealing for toxicology laboratories due to the quick extraction, minimal amounts of sample and solvents used, and more than 100 possible reutilizations of the mixed mode sorbent.

To the best of our knowledge, this is the first method to use MEPS in combination with GC-MS to identify and quantify the main cannabinoids in urine.

## Figures and Tables

**Figure 6:**
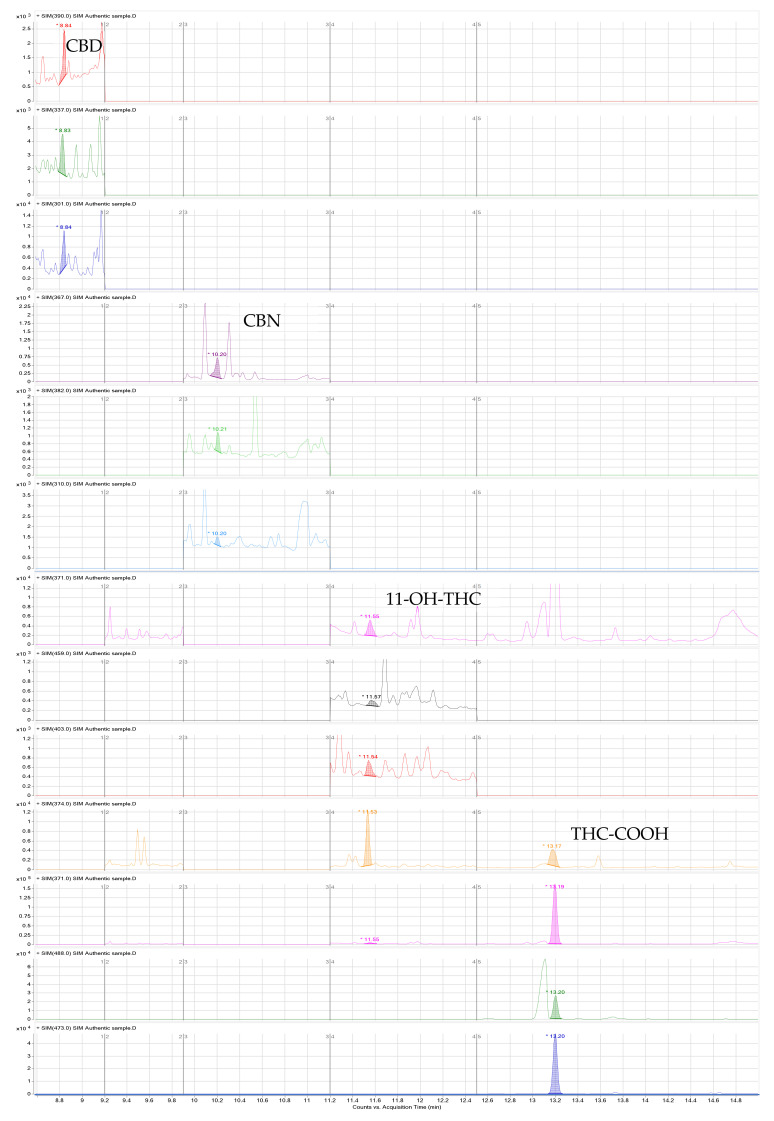
Chromatogram of an authentic sample (number 6 in Table 6).

**Table 1:** Linearity data (n = 5).

Compound	Weight	Linear Range (ng/mL)	Linearity		R^2^ ª	LOD (ng/mL)	LLOQ (ng/mL)
			Slope ª	Intercept ª			
CBD	1/x	1–400	0.1118 ± 0.0136	2.9449 ± 4.0245	0.9980 ± 0.0014	1	1
THC		1–400	0.0488 ± 0.0089	0.6771 ± 0.5143	0.9990 ± 0.0008	1	1
CBN		5–400	0.2865 ± 0.0275	0.1373 ± 0.5117	0.9967 ± 0.0030	5	5
11-OH-THC		5–400	0.0678 ± 0.0033	0.1173 ± 0.1607	0.9983 ± 0.0012	1	5
THC-COOH		10–400	0.0588 ± 0.0065	0.3577 ± 0.2330	0.9984 ± 0.0010	5	10

ª Mean ± standard deviation.

**Table 2:** Comparison of limits of detection and quantification of several methods.

Analyte	Sample Amount (mL)	LOD (ng/mL)	LLOQ (ng/mL)	Method of Detection	Reference
CBD	1.00	1.00	ns	LC-MS/MS	[38]
THC		1.00			
CBN		1.00			
11-OH-THC		1.00			
THC-COOH		5.00			
CBD	0.09	3.00	10.00	LC-MS/MS	[39]
THC		3.00	8.00		
CBN		4.00 *	9.00		
11-OH-THC		3.00	9.00		
THC-COOH		2.00 *	6.00 *		
CBD	1.00	3.00	9.00	LC-MS/MS	[40]
THC		2.00	8.00		
CBN		4.00 *	12.00		
11-OH-THC		2.00 *	6.00		
THC-COOH		2.00 *	6.00 *		
CBD	2.00	5.00	16.00	GC-MS	[41]
THC		3.00	9.00		
CBN		5.00	18.00		
11-OH-THC		2.60 *	8.70		
THC-COOH		4.50 *	15.00		
CBD	0.20	2.00	**	LC-MS/MS	[42]
THC		1.00			
CBN		2.00 *			
11-OH-THC		2.00			
THC-COOH		1.00 *			
THC	2.00	2.50	3.00	GC-MS	[43]
11-OH-THC		1.00	2.00 *		
THC-COOH		1.00 *	2.00 *		
THC	2.00	1.00	2.50	GC-MS	[44]
11-OH-THC		2.50	2.50 *		
THC-COOH		2.50 *	2.50 *		
THC	1.00	5.00	20.00	LC-MS/MS	[33]
11-OH-THC		5.00	20.00		
THC-COOH		1.00 *	5.00 *		
THC	0.50	1.00	2.50	LC-MS/MS	[45]
THC-COOH		1.00 *	2.50 *		
THC-COOH	0.50	5.00	10.00	LC-MS/MS	[46]
THC-COOH	0.50	5.00	10.00	LC-MS/MS	[47]
CBD	0.25	ns	10.00	LC-MS/MS	[48]
THC			10.00		
CBN			10.00		
11-OH-THC			10.00		
THC-COOH			10.00		
CBD	0.025	0.30	10.00	LC-MS/MS	[49]
THC		0.30	10.00		
CBN		1.40	10.00		
11-OH-THC		1.90	10.00		
THC-COOH		1.40	10.00		
	THC-COOH	0.50 *	1.00 *	GC-MS/MS	[50]
CBD	0.25	0.10 *	0.30 *	GC-MS/MS	[51]
THC		0.15 *	0.30 *		
CBN		0.15 *	0.20 *		
11-OH-THC		0.15 *	0.30 *		
THC-COOH		1.00 *	3.00 *		
CBD	1.00	0.20 *	0.30 *	GC-MS/MS	[52]
THC		0.20 *	0.30 *		
CBN		0.20 *	0.30 *		
11-OH-THC		0.20 *	0.30 *		
THC-COOH		2.00 *	3.00 *		
THC-COOH	ns	1.00 *	5.00 *	GC-MS/MS	[53]
THC	1.50	0.78 *	**	UHPLC-MS/MS	[54]
11-OH-THC		0.78 *			
THC-COOH		0.78 *			
CBD	0.20	0.50 *	**	LC-MS/MS	[55]
THC		0.50 *			
CBN		0.50 *			
11-OH-THC		0.50 *			
THC-COOH		0.50 *			
THC-COOH	0.12	0.20 *	0.70 *	LC-MS/MS	[56]
THC-COOH	1.00	0.20 *	5.00 *	LC-MS/MS	[59]
THC	0.10–1.00	0.16 *	0.27 *	LC-MS/MS	[58]
11-OH-THC		0.17 *	0.28 *		
THC-COOH		0.14 *	0.23 *		
THC-COOH	0.20	0.50 *	7.50 *	LC-MS/MS	[57]
CBD	10.00	0.20 *	1.00	HPLC-UV	[60]
THC		0.50 *	1.00		
CBN		0.10 *	1.00 *		

* lower limits than the present work; ** LOD values are the same as LLOQ; ns: not specified.

**Table 3:** Inter-day, intraday, and intermediate precision and accuracy.

Analyte	Concentration (ng/mL)	Inter-Day (n = 5)		Intra-Day (n = 5)		Intermediate (n = 15)	
		CV (%)	RE (%)	CV (%)	RE (%)	CV (%)	RE (%)
CBD	1	10.27	6.68	7.67	7.38		
	5	14.26	1.80	10.50	9.06		
	10	9.02	6.34				
	15			10.08	3.04	2.80	1.46
	50	1.04	1.04				
	100	3.13	3.13	9.10	9.55		
	200	1.68	1.68				
	240					9.12	0.43
	300	3.51	3.51				
	360					7.84	4.87
	400	0.49	0.49	9.81	6.98		
THC	1	8.71	0.88	7.27	3.94		
	5	9.23	0.76	5.00	7.51		
	10	9.61	1.39				
	15			9.03	0.76	7.01	3.91
	50	10.00	2.89				
	100	4.49	1.39	9.56	8.52		
	200	2.89	0.79				
	240					7.14	1.35
	300	1.75	1.10				
	360					5.84	3.53
	400	1.45	0.79	5.08	7.39		
CBN	5	9.39	4.17	2.02	11.37		
	10	7.37	0.74				
	15			14.57	6.36	11.71	1.78
	50	0.40	14.69				
	100	0.21	5.01	8.57	9.38		
	200	6.08	0.15				
	240					8.08	6.95
	300	6.44	2.87				
	360					9.52	9.21
	400	2.55	2.61	14.72	5.05		
11-OH-THC	5	4.54	9.68	3.21	9.83		
	10	5.60	1.41				
	15			5.62	2.68	12.11	1.39
	50	6.33	8.67				
	100	2.86	13.80	2.24	0.81		
	200	1.91	0.93				
	240					9.99	2.61
	300	1.01	0.23				
	360					10.52	3.77
	400	2.06	1.34	12.92	1.24		
THC-COOH	10	10.15	3.07	1.48	14.00		
	15			12.80	1.77	8.15	4.74
	50	13.26	13.26				
	100	14.41	14.41	3.55	0.08		
	200	1.09	1.15				
	240					10.95	0.86
	300	1.71	0.37				
	360					7.92	5.95
	400	1.45	0.36	13.53	0.94		

CV: coefficient of variation; RE: relative error.

**Table 4:** Extraction efficiency (%) of the target analytes (n = 3).

Analyte	Recovery (%) ª		
	50 ng/mL	100 ng/mL	400 ng/mL
CBD	37.9 ± 3.8	42.6 ± 8.1	51.1 ± 7.6
THC	28.0 ± 2.9	30.6 ± 5.8	26.7 ± 3.8
CBN	30.7 ± 3.5	33.7 ± 5.7	57.3 ± 4.7
11-OH-THC	47.7 ± 6.3	66.5 ± 7.2	74.7 ± 12.6
THC-COOH	63.4 ± 12.1	85.8 ± 11.5	82.6 ± 14.9

ª Mean ± standard deviation.

**Table 5:** Stability evaluation.

Analyte	Concentration (ng/mL)	Processed Samples (n = 3)		Shor-Term Stability (n = 3)		Freeze/Thaw Stability (n = 3)	
		CV (%)	RE (%)	CV (%)	RE (%)	CV (%)	RE (%)
CBD	15	1.64	5.01	1.83	10.62	1.69	11.82
	240	13.36	13.32	1.85	0.27	1.00	14.02
	360	5.48	0.04	9.22	0.89	3.14	12.45
THC	15	0.33	13.40	2.28	12.27	1.95	5.93
	240	2.09	9.09	9.06	6.91	6.45	5.70
	360	3.91	2.29	6.97	14.36	1.55	3.46
CBN	15	12.06	3.07	0.86	13.71	0.41	4.89
	240	9,87	1.59	0.50	13.54	6.36	9.39
	360	7.29	2.26	0.18	14.52	3.45	8.74
11-OH-THC	15	5.14	4.29	0.42	11.98	5.69	6.15
	240	6.65	5.69	6.03	7.62	1.81	13.23
	360	5.45	2.86	5.67	5.05	1.83	2.27
THC-COOH	15	14.20	2.70	2.41	12.66	0.21	13.55
	240	0.12	13.10	0.05	5.90	9.84	2.06
	360	1.43	0.96	1.43	13.69	6.42	7.92

CV: coefficient of variation; RE: relative error.

**Table 6:** Concentrations of cannabinoids found in authentic urine samples.

Sample Number	Concentration (ng/mL)				
	CBD	THC	CBN	11-OH-THC	THC-COOH
1	Negative	Negative	4.84	0.04	115.61
2	Negative	Negative	6.58	0.30	90.32
3	0.29	Negative	5.01	0.53	63.56
4	8.36	Negative	5.11	0.60	21.89
5	1.86	Negative	4.70	0.43	29.59
6	1.96	Negative	2.03	5.54	556.18
7	0.90	Negative	1.00	3.58	6.66

A chromatogram of an authentic sample (number 6) is shown in Figure 6.

**Table 7:** Retention times and selected transitions for the identification of analytes.

**Analyte**	**Retention Time (min)**	**Ions (*m*/*z*)**	**Dwell Time (µs)**
CBD		390	100
	8.84	337	100
		301	100
THC	9.57	371	100
		386	100
		315	100
THC-d_3_ ª	9.53	374	50
CBN	10.20	367	100
		382	100
		310	100
11-THC-OH	11.56	371	100
		449	100
		403	100
11-THC-OH-d_3_ ª	11.54	374	50
THC-COOH	13.20	371	100
		488	100
		473	100
THC-COOH-d_3_ ª	13.17	374	50

ª internal standard; quantifying ions underlined.

## Data Availability

Not applicable.

## References

[1] Cannabis: Health and Social Responses. https://www.emcdda.europa.eu/publications/mini-guides/cannabis-health-and-social-responses_en.

[2] Gonçalves J., Rosado T., Soares S., Simão A., Caramelo D., Luís Â., Fernández N., Barroso M., Gallardo E., Duarte A. (2019). Cannabis and Its Secondary Metabolites: Their Use as Therapeutic Drugs, Toxicological Aspects, and Analytical Determination. Medicines.

[3] (2021). United Nations Office on Drugs and Crime (ONUDC) Global Overview: Drug Demand.

[4] European Monitoring Centre for Drugs and Drug Addiction (EMCDDA) (2018). Cannabis Legislation in EUROPE.

[5] Preuss U.W., Huestis M.A., Schneider M., Hermann D., Lutz B., Hasan A., Kambeitz J., Wong J.W.M., Hoch E. (2021). Cannabis Use and Car Crashes: A Review. Front. Psychiatry.

[6] Molinaro S., Vicente J., Benedetti E., Cerrai S., Colasante E., Arpa S., Chomynová P., Kraus L., Monshouwer K. (2019). ESPAD Report.

[7] European Monitoring Centre for Drugs and Drug Addiction European Drug Report: Trends and Developments; 2021. https://www.emcdda.europa.eu/publications/edr/trends-developments/2021_en.

[8] Matheson J., Mann R.E., Sproule B., Huestis M.A., Wickens C.M., Stoduto G., George T.P., Rehm J., Le Foll B., Brands B. (2020). Acute and residual mood and cognitive performance of young adults following smoked cannabis. Pharmacol. Biochem. Behav..

[9] Wickens C.M., Wright M., Mann R.E., Brands B., Di Ciano P., Stoduto G., Fares A., Matheson J., George T.P., Rehm J. (2022). Separate and combined effects of alcohol and cannabis on mood, subjective experience, cognition and psychomotor performance: A randomized trial. Prog. Neuropsychopharmacol. Biol. Psychiatry.

[10] Kancherla N., Jeyanthi K., Abbas R., Sathi T., Upadhyay A., Garlapati S. (2021). Cannabis Associated Mental Health Effects: A Review. J. Pharm. Bioallied Sci..

[11] Bonini S.A., Premoli M., Tambaro S., Kumar A., Maccarinelli G., Memo M., Mastinu A. (2018). Cannabis sativa: A comprehensive ethnopharmacological review of a medicinal plant with a long history. J. Ethnopharmacol..

[12] Legare C.A., Raup-Konsavage W.M., Vrana K.E. (2022). Therapeutic Potential of Cannabis, Cannabidiol, and Cannabinoid-Based Pharmaceuticals. Pharmacology.

[13] ElSohly M.A., Radwan M.M., Gul W., Chandra S., Galal A. (2017). Phytochemistry of *Cannabis sativa* L. Progress in the Chemistry of Organic Natural Products.

[14] Filipiuc L.E., Ababei D.C., Alexa-Stratulat T., Pricope C.V., Bild V., Stefanescu R., Stanciu G.D., Tamba B.I. (2021). Major phytocannabinoids and their related compounds: Should we only search for drugs that act on cannabinoid receptors?. Pharmaceutics.

[15] Lucas C.J., Galettis P., Schneider J. (2018). The pharmacokinetics and the pharmacodynamics of cannabinoids. Br. J. Clin. Pharmacol..

[16] Karschner E.L., Swortwood-Gates M.J., Huestis M.A. (2020). Identifying and Quantifying Cannabinoids in Biological Matrices in the Medical and Legal Cannabis Era. Clin. Chem..

[17] D’Orazio A.L., Mohr A.L.A., Chan-Hosokawa A., Harper C., Huestis M.A., Limoges J.F., Miles A.K., Scarneo C.E., Kerrigan S., Liddicoat L.J. (2021). Recommendations for Toxicological Investigation of Drug-Impaired Driving and Motor Vehicle Fatalities-2021 Update. J. Anal. Toxicol..

[18] Abdel-Rehim M. (2011). Microextraction by packed sorbent (MEPS): A tutorial. Anal. Chim. Acta.

[19] Moein M.M., Abdel-Rehim A., Abdel-Rehim M. (2015). Microextraction by packed sorbent (MEPS). TrAC Trends Anal. Chem..

[20] Rosado T., Gallardo E., Vieira D.N., Barroso M. (2021). Microextraction by Packed Sorbent. Microextraction Techniques in Analytical Toxicology.

[21] Abdel-Rehim M. (2010). Recent advances in microextraction by packed sorbent for bioanalysis. J. Chromatogr. A.

[22] Santos C., Oppolzer D., Gonçalves A., Barroso M., Gallardo E. (2018). Determination of Organophosphorous Pesticides in Blood Using Microextraction in Packed Sorbent and Gas Chromatography-Tandem Mass Spectrometry. J. Anal. Toxicol..

[23] Rosado T., Gallardo E., Vieira D.N., Barroso M. (2020). Microextraction by Packed Sorbent as a Novel Strategy for Sample Clean-Up in the Determination of Methadone and EDDP in Hair. J. Anal. Toxicol..

[24] Rosado T., Gonçalves A., Margalho C., Barroso M., Gallardo E. (2017). Rapid analysis of cocaine and metabolites in urine using microextraction in packed sorbent and GC/MS. Anal. Bioanal. Chem..

[25] Malaca S., Rosado T., Restolho J., Rodilla J.M., Rocha P.M.M., Silva L., Margalho C., Barroso M., Gallardo E. (2019). Determination of amphetamine-type stimulants in urine samples using microextraction by packed sorbent and gas chromatography-mass spectrometry. J. Chromatogr. B Analyt. Technol. Biomed. Life Sci..

[26] Simão A.Y., Monteiro C., Marques H., Rosado T., Margalho C., Barroso M., Andraus M., Gallardo E. (2022). Analysis of opiates in urine using microextraction by packed sorbent and gas Chromatography-Tandem mass spectrometry. J. Chromatogr. B.

[27] Sorribes-Soriano A., Verdeguer J., Pastor A., Armenta S., Esteve-Turrillas F.A. (2021). Determination of Third-Generation Synthetic Cannabinoids in Oral Fluids. J. Anal. Toxicol..

[28] Pautova A.K., Khesina Z.B., Litvinova T.N., Revelsky A.I., Beloborodova N.V. (2021). Metabolic profiling of aromatic compounds in cerebrospinal fluid of neurosurgical patients using microextraction by packed sorbent and liquid–liquid extraction with gas chromatography–mass spectrometry analysis. Biomed. Chromatogr..

[29] Abdel-Rehim M. (2004). New trend in sample preparation: On-line microextraction in packed syringe for liquid and gas chromatography applications. J. Chromatogr. B.

[30] Silva C., Cavaco C., Perestrelo R., Pereira J., Câmara J. (2014). Microextraction by Packed Sorbent (MEPS) and Solid-Phase Microextraction (SPME) as Sample Preparation Procedures for the Metabolomic Profiling of Urine. Metabolites.

[31] Sergi M., Montesano C., Odoardi S., Mainero Rocca L., Fabrizi G., Compagnone D., Curini R. (2013). Micro extraction by packed sorbent coupled to liquid chromatography tandem mass spectrometry for the rapid and sensitive determination of cannabinoids in oral fluids. J. Chromatogr. A.

[32] Rosado T., Fernandes L., Barroso M., Gallardo E. (2017). Sensitive determination of THC and main metabolites in human plasma by means of microextraction in packed sorbent and gas chromatography–tandem mass spectrometry. J. Chromatogr. B.

[33] Sartore D.M., Vargas Medina D.A., Costa J.L., Lanças F.M., Santos-Neto Á.J. (2020). Automated microextraction by packed sorbent of cannabinoids from human urine using a lab-made device packed with molecularly imprinted polymer. Talanta.

[34] Scientific Working Group for Forensic Toxicology (SWGTOX) (2013). Standard Practices for Method Validation in Forensic Toxicology. J. Anal. Toxicol..

[35] Food and Drug Administration Bioanalytical Method Validation Guidance for Industry. https://www.fda.gov/regulatory-information/search-fda-guidance-documents/bioanalytical-method-validation-guidance-industry.

[36] Substance Abuse and Mental Health Services Administration (SAMHSA) (2012). Clinical Drug Testing in Primary Care. Technical Assistance Publication Series.

[37] European Workplace Drug Testing Society (2015). European Guidelines for Workplace Drug Testing in Urine 2015-11-01.

[38] Reber J.D., Karschner E.L., Seither J.Z., Knittel J.L., Walterscheid J.P. (2021). Screening and confirmation methods for the qualitative identification of nine phytocannabinoids in urine by LC-MS/MS. Clin. Biochem..

[39] Montesano C., Sergi M., Odoardi S., Simeoni M.C., Compagnone D., Curini R. (2014). A μ-SPE procedure for the determination of cannabinoids and their metabolites in urine by LC–MS/MS. J. Pharm. Biomed. Anal..

[40] Jagerdeo E., Montgomery M.A., Karas R.P., Sibum M. (2010). A fast method for screening and/or quantitation of tetrahydrocannabinol and metabolites in urine by automated SPE/LC/MS/MS. Anal. Bioanal. Chem..

[41] Korac N., Vidic D., Sutlović D. (2020). Modified QuEChERS extraction and GC-MS analysis of selected cannabinoids from human urine. Glas. Hem. Tehnol. Bosne Hercegovine.

[42] Sempio C., Scheidweiler K.B., Barnes A.J., Huestis M.A. (2018). Optimization of recombinant β-glucuronidase hydrolysis and quantification of eight urinary cannabinoids and metabolites by liquid chromatography tandem mass spectrometry. Drug Test. Anal..

[43] Nestić M., Babić S., Pavlović D.M., Sutlović D. (2013). Molecularly imprinted solid phase extraction for simultaneous determination of δ9-tetrahydrocannabinol and its main metabolites by gas chromatography-mass spectrometry in urine samples. Forensic Sci. Int..

[44] Abraham T.T., Lowe R.H., Pirnay S.O., Darwin W.D., Huestis M.A. (2007). Simultaneous GC-EI-MS determination of Δ9-tetrahydrocannabinol, 11-hydroxy-Δ9-tetrahydrocannabinol, and 11-nor-9-carboxy-Δ9-tetrahydrocannabinol in human urine following tandem enzyme-alkaline hydrolysis. J. Anal. Toxicol..

[45] Lendoiro E., De Castro A., Fernández-Vega H., Cela-Pérez M.C., López-Vilariño J.M., González-Rodríguez M.V., Cruz A., López-Rivadulla M. (2014). Molecularly imprinted polymer for selective determination of Δ9-tetrahydrocannabinol and 11-nor-Δ9-tetrahydrocannabinol carboxylic acid using LC-MS/MS in urine and oral fluid Forensic Toxicology. Anal. Bioanal. Chem..

[46] Rumpler M.J. (2014). Quantitative analysis of 11-nor-9-carboxy-tetrahydrocannbinol (THC-COOH) in urine by LC-MS/MS following a simple filtration. J. Chromatogr. B Anal. Technol. Biomed. Life Sci..

[47] Felli M., Martello S., Chiarotti M. (2011). LC-MS-MS method for simultaneous determination of THCCOOH and THCCOOH-glucuronide in urine: Application to workplace confirmation tests. Forensic Sci. Int..

[48] Morisue Sartore D., Costa J.L., Lanças F.M., Santos-Neto Á.J. (2022). Packed in-tube SPME–LC–MS/MS for fast and straightforward analysis of cannabinoids and metabolites in human urine. Electrophoresis.

[49] Moretti M., Freni F., Carelli C., Previderé C., Grignani P., Vignali C., Cobo-Golpe M., Morini L. (2021). Analysis of Cannabinoids and Metabolites in Dried Urine Spots (DUS). Molecules.

[50] De M Prata V., Emídio E.S., Dorea H.S. (2012). New catalytic ultrasound method for derivatization of 11-nor-Δ9-tetrahydrocannabinol-9-carboxylic acid in urine, with analysis by GC-MS/MS. Anal. Bioanal. Chem..

[51] Frei P., Frauchiger S., Scheurer E., Mercer-Chalmers-Bender K. (2022). Quantitative determination of five cannabinoids in blood and urine by gas chromatography tandem mass spectrometry applying automated on-line solid phase extraction. Drug Test. Anal..

[52] Meier U., Dussy F., Scheurer E., Mercer-Chalmers-Bender K., Hangartner S. (2018). Cannabinoid concentrations in blood and urine after smoking cannabidiol joints. Forensic Sci. Int..

[53] Rodrigues L.C., Kahl J.M., de Chinaglia K.O., de Campos E.G., Costa J.L. (2022). Dispersive liquid–liquid microextraction of 11-nor-Δ9-tetrahydrocannabinol-carboxylic acid applied to urine testing. Bioanalysis.

[54] Saenz S.R., Lewis R.J., Angier M.K., Wagner J.R. (2017). Postmortem Fluid and Tissue Concentrations of THC, 11-OH-THC and THC-COOH^†^. J. Anal. Toxicol..

[55] Andersson M., Scheidweiler K.B., Sempio C., Barnes A.J., Huestis M.A. (2016). Simultaneous quantification of 11 cannabinoids and metabolites in human urine by liquid chromatography tandem mass spectrometry using WAX-S tips. Anal. Bioanal. Chem..

[56] Stephanson N., Josefsson M., Kronstrand R., Beck O. (2008). Accurate identification and quantification of 11-nor-Δ9-tetrahydrocannabinol-9-carboxylic acid in urine drug testing: Evaluation of a direct high efficiency liquid chromatographic-mass spectrometric method. J. Chromatogr. B Anal. Technol. Biomed. Life Sci..

[57] Chebbah C., Pozo O.J., Deventer K., Van Eenoo P., Delbeke F.T. (2010). Direct quantification of 11-nor-Δ 9 -tetrahydrocannabinol-9-carboxylic acid in urine by liquid chromatography/tandem mass spectrometry in relation to doping control analysis. Rapid Commun. Mass Spectrom..

[58] Sánchez-González J., Salgueiro-Fernández R., Cabarcos P., Bermejo A.M., Bermejo-Barrera P., Moreda-Piñeiro A. (2017). Cannabinoids assessment in plasma and urine by high performance liquid chromatography–tandem mass spectrometry after molecularly imprinted polymer microsolid-phase extraction. Anal. Bioanal. Chem..

[59] Zanchetti G., Floris I., Piccinotti A., Tameni S., Polettini A. (2012). Rapid and robust confirmation and quantification of 11-nor-Δ9-tetrahydrocannabinol-9-carboxylic acid (THC-COOH) in urine by column switching LC-MS-MS analysis. J. Mass Spectrom..

[60] Moradi M., Yamini Y., Baheri T. (2011). Analysis of abuse drugs in urine using surfactant-assisted dispersive liquid-liquid microextraction. J. Sep. Sci..

[61] Pena-Pereira F., Wojnowski W., Tobiszewski M. (2020). AGREE—Analytical GREEnness Metric Approach and Software. Anal. Chem..

[62] Wojciech Wojnowski—Science Profile—MOST Wiedzy. https://mostwiedzy.pl/en/wojciech-wojnowski,174235-1/AGREE?.

[63] Schroeder J.L., Marinetti L.J., Smith R.K., Brewer W.E., Clelland B.L., Morgan S.L. (2008). The analysis of Δ9-tetrahydrocannabinol and metabolite in whole blood and 11-nor-Δ9-tetrahydrocannabinol-9-carboxylic acid in urine using disposable pipette extraction with confirmation and quantification by gas chromatography-mass spectrometry. J. Anal. Toxicol..

[64] Kramer K.E., Andrews A.R.J. (2001). Screening method for 11-nor-Δ9-tetrahydrocannabinol-9-carboxylic acid in urine using hollow fiber membrane solvent microextraction with in-tube derivatization. J. Chromatogr. B Biomed. Sci. Appl..

[65] At a Glance—Estimates of Drug Use in The European Union (Updated June 2021). https://www.emcdda.europa.eu/media-library/glance---estimates-drug-use-european-union-updated-june-2021_en.

